# Rational Design of Waterborne Polyurethane Pressure Sensitive Adhesives for Different Working Temperatures

**DOI:** 10.3390/ma15062011

**Published:** 2022-03-08

**Authors:** Hui Zhao, Ying Xu, Zhen Luo, Cui-Ran Gong, Yang-Qing Zheng, Li-Ming Yu

**Affiliations:** 1 College of Chemistry & Materials Science, Fujian Normal University, Fuzhou 350007, China; huizhao@fjirsm.ac.cn; 2 Key Laboratory of Coal to Ethylene Glycol and Its Related Technology, Fujian Institute of Research on the Structure of Matter, Chinese Academy of Sciences, Fuzhou 350000, China; zhenluo@fjirsm.ac.cn (Z.L.); gongcuiran@163.com (C.-R.G.); yqzheng@fjirsm.ac.cn (Y.-Q.Z.); yuliming@fjirsm.ac.cn (L.-M.Y.)

**Keywords:** waterborne polyurethane (WPU), pressure sensitive adhesive (PSA), rheology, adhesive performance, rational design

## Abstract

The appropriate pressure sensitive adhesion performances at working temperature are vital for the applications of waterborne polyurethane (WPU). Understanding the relationship among rheological behaviors, macromolecular structures and adhesive performances can be very useful to the rational design of waterborne polyurethane pressure sensitive adhesives (WPU-PSAs) for different operating temperatures, as well as other kinds of adhesives. In this study, four kinds of WPU-PSAs were prepared by reacting polypropylene glycol (PPG), hydrogenated hydroxyl-terminated polybutadiene (HHTPB), dimethyl alcohol propionic acid (DMPA), 1,6-hexamethylene diisocyanate (HDI) and four kinds of chain extenders. Gel permeation chromatography (GPC), swelling and rheology tests were used in parallel with an analysis of adhesive performances of the dried films of the adhesives. Results showed that, in addition to the nature of chain extenders playing a role on the rheological behaviors and adhesive performances of polymer, the gel content could be used to adjust the macromolecular structure and molecular weight distribution of polymer, thus distinctly affected the adhesive performances of PSA. The relationship among rheological behaviors, macromolecular structure and adhesive performances was investigated, and the rational design of WPU was achieved with appropriate pressure sensitive adhesion properties for different working temperatures of 25 and 60 °C.

## 1. Introduction

Pressure sensitive adhesive (PSA) is a class of self-adhesive material. Under the extrusion of a small external force, PSA could form good contact with most substrates [1]. The adhesive performances of PSA generally refer to tack, peel and holding power. Among the three performances, tack is the most special. Tack is the instantaneous bonding performances of an adhesive under the application of light pressure. In fact, tack is a performance of all adhesives, however, almost all the studies on the tack have been carried out only on PSA, as it is mostly obvious and essential for PSA [2,3,4,5,6,7]. Tack is characterized by the force required to separate a specific area of an adhesive from an adherend, which is partly similar to the characterization of peel, however, a peel test is corresponded to a high frequency operation [8,9]. Holding power is the performance of shear resistance of an adhesive. All the three adhesive performances are complex properties affected by the temperature, adhesive itself, adhesion interface and testing procedure, nevertheless, a general trend has been discovered in the studies of PSA. It is found that high performance PSA must have a proper combinational property of both liquid-like (viscous part) and solid-like (elastic part), and the liquid-like means a low modulus of elasticity and a high dissipation of viscosity, whereas the solid-like means a high modulus of elasticity and a low dissipation [8,10]. Different balances between the liquid-like component and solid-like component can lead to different PSA performances [11,12,13,14,15,16,17].

On the other hand, in consideration of environmental protection, works on waterborne polyurethane pressure sensitive adhesives (WPU-PSAs) have been carried out in recent years [18,19,20]. What is more, the flexible adjustment of polymeric chain segments of WPU also provides a means to control the molecular structure of polymer and the composition of adhesive film on a micro scale. Polymers with optimized physically or chemically cross-linked structure can offer relatively high-performance PSA [3,11,14]. Many works have been focused on the preparation of general WPU-PSAs by adjusting the crosslinking of WPU [2,4,19]. The cross-linked structures not only changed hydrogen bonds content in the polymer, but also led to a wider molecular weight distribution of the polymer, which was closely related to adhesive performances of a PSA [21]. An efficient acrylic/PU hybrid system was developed to control finely the structure networks of polymer and affected adhesive performances [22,23]. Different types of WPU-PSAs (removable, high shear resistance) were achieved, attributed to the different ratio of polyurethane and polyurea by changing the number of hydroxyl groups in the amino-alcohol chain extender and hence of different levels of crosslinking [4]. Both tack and peel strength of WPU-PSAs were increased by a small addition of polycarbodiimide as crosslinking agents [15]. Nevertheless, due to the complexity of chemical reactions of polyurethane, the fine control of the crosslinking components and the effects of the crosslinking content on the adhesive performances have been far from being exploited fully in WPU-PSAs.

WPU has been widely used in the industry, such as adhesives, inks and coatings [4,24,25,26,27]. Pressure sensitive adhesion properties at different working temperatures are vital for the practical usages of WPU [13], as appropriate tack performance and other adhesive performances are required to adapt to different manufacturing processes. For example, sole adhesive must have a good tack at approximately 60 °C, whereas counterparts for the rolling combination of some artificial leather and cloth are at approximately 120 °C. WPU is used on various materials and various operating temperatures, thus PSA properties of WPU at the working temperature rather than at the room temperature are of particular concern. However, at non-room temperature, the pressure sensitive adhesion properties of WPU are often neglected and rarely researched [13,28]. In order to promote the further development of WPU, it is necessary to investigate and reasonably design the adhesive performances of WPU-PSAs for different working temperatures.

Current preparations of WPU are mainly conducted by adjusting the formula, and the approach to obtain the target product is still trial and error. Moreover, more trials and errors are often required to obtain an appropriate WPU that binds at a non-room temperature. Fortunately, the rheology behavior of WPU macromolecules is like a bridge linked between the micro molecular structures of polymer and the macro PSA properties, which could help reducing costly trial and error from manufacturing from a new perspective point to understand and modify the properties of WPU. Unlike previous reports, our work is focused on how the three adhesive properties vary with temperature, and correlating the variation to the rheological behavior and structure of WPU macromolecules. The PSA properties versus temperature can be anticipated to a certain extent by dynamic modulus and other parameters versus temperature or frequency, and correspondingly, both adhesion success and adhesion failure could be analyzed reasonably and designed.

Here, a series of WPU-PSAs with four kinds of extenders were synthesized, and the relationship among their structures, rheological behaviors and adhesive performances was investigated. Gel permeation chromatography (GPC), swelling and rheology tests were used in parallel with determinations of macroscopic adhesive performances of the dried films at different temperatures. Results showed that, in addition to the nature of chain extenders playing a role on both the rheological behaviors and adhesive performances of polymer, the gel content could be used to adjust the molecular structure and molecular weight distribution of polymer, thus distinctly affected the rheological behaviors and adhesive performances of WPU-PSA. The problems of heat-resistance and adhesive residue of PSAs were also of concern, and the solutions were put forward. The rational design of WPU with proper pressure sensitive adhesion performances for different working temperatures was attempted significantly by using four kinds of chain extenders. By deciphering the contributions of the chain extenders into the adhesive performances changes at temperature of 25 and 60 °C of four WPU-PSAs, this study would be carried out on how the three adhesive properties of PSA vary with temperature could be correlated to the rheological behaviors and molecular structures. To the best of our knowledge, there were very few reports dedicated to the pressure sensitive adhesion properties of WPU at different temperatures, and only the tack property at the highest temperature of 37 °C had been studied. Although all the three adhesive properties of PSA vary with temperature, there is no report on the relationship between the variation of all the three adhesive properties and rheological properties and macromolecular structures of an adhesive.

## 2. Experimental Section

### 2.1. Materials

**Figure 1:**
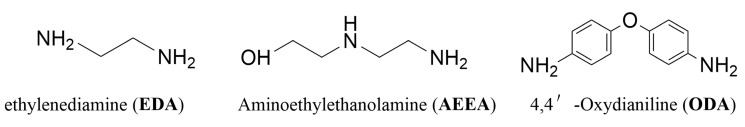
Chemical structures of amine chain extenders.

Polypropylene glycol with molecular weight of 2000 Da (PPG2000) supplied by Sinopharm (Shanghai, China) and Hydrogenated hydroxyl-terminated polybutadiene with molecular weight of 2000 Da (HHTPB2000) supplied by Total Cray Valley (Paris, France ) were used as polyols. Hexamethylene diisocyanate (HDI) supplied by Covestro (Shanghai, China) was used as the aliphatic isocyanate. Dibutyltin dilaurate (DBTDL) supplied by Sinopharm (Shanghai, China) was used as the catalyst. 2,2′-Bis(hydroxymethyl) propionic (DMPA) from MACKLIN (Shanghai, China) was used as a potential hydrophilic agent. Both triethylamine (TEA) and methyl ethyl ketone (MEK) were purchased from Sinopharm (Shanghai, China). The chain extenders were 1,4-butanediol (BDO) and ethylenediamine (EDA) provided by Sinopharm (Shanghai, China), and Aminoethyl ethanolamine (AEEA) and 4,4′-Diaminodiphenyl ether (ODA) provided by MACKLIN (Shanghai, China), respectively. The chemical structures of the three amine chain extenders were shown in Figure 1.

### 2.2. Syntheses of WPU-PSAs

**Figure 2:**
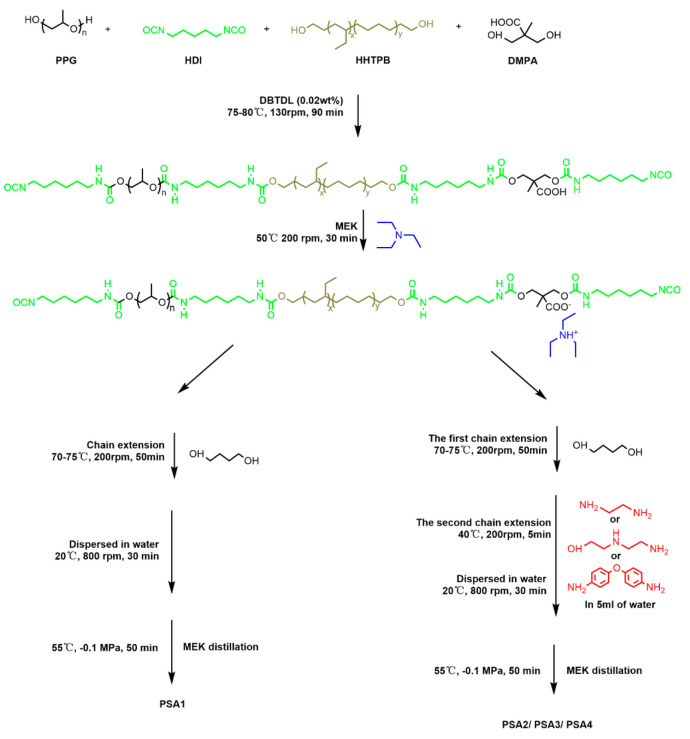
Schematic synthesis of waterborne polyurethane pressure sensitive adhesives (WPU-PSAs).

The mixture of PPG (22.5 g, 11.25 mmol), HHTPB (2.5 g, 1.25 mmol) and DMPA (1.675 g, 12.5 mmol) was dehydrated under reduced pressure (−0.1 MPa) at 110 °C for 1 h then cooled to 75 °C. HDI (6.307, 37.5 mmol) and 0.02 wt% of DBTDL were added with a stirring speed of 130 rpm for 90 min at 75–80 °C. Then, MEK (28 mL) was used to adjust the viscosity of the prepolymer, TEA (1.265 g, 12.5 mmol) was added to neutralize the carboxyl groups to obtain hydrophilic groups on the prepolymer chains. The neutralization process lasted 30 min at 50 °C. Then, two sets of preparation processes were used to achieve the chain growth of prepolymer. The first set was that a certain amount of BDO (1.127 g, 12.5 mmol) was added and stirred at 70–75 °C for 50 min, then, the required amount of water was added and stirred gradually to a high speed of 800 rpm and maintained at 20 °C for 50 min. The second set was that less amount of BDO (0.902 g, 10 mmol) was added and stirred at 75 °C for 50 min, then, water solution (5 mL) of an amine (2.5 mmol) was added and stirred at 40 °C for 5 min, then, the required amount of water was added and stirred gradually to a high speed of 800 rpm and maintained at 55 °C for 50 min. After the MEK had been removed by vacuum distillation, polyurethane dispersions were obtained with a solid content of approximately 22%. Four WPU-PSAs were obtained, and the syntheses process was shown in Figure 2. The four WPU-PSAs were prepared with four kinds of chain extenders, respectively, and the compositions were listed in Table 1. For the sample codes, PSA1 denoted the WPU-PSAs with BDO as the chain extender, PSA2 represented that with both BDO and EDA as the chain extender, PSA3 represented that with both BDO and AEEA as the chain extender, and PSA4 represented that with both BDO and ODA as the chain extender.

## 3. Characterization

Attenuated total reflection Fourier transformed infrared spectroscopy (ATR−FTIR) of the fours WPU-PSAs were characterized by Thermo Fisher Scientific NICOLET iS 5 (Thermo Fisher Scientific, Waltham, MA, USA). The spectra of the samples were obtained after 16 scans between 4000 and 400 cm^−1^.

The thermal gravimetric analysis (TGA) of four WPU-PSAs was measured by Netzsch STA449F3 (NETZSCH Instrument Manufacturing GmbH, Selb, Germany). Dried film of approximately 5 mg of WPU-PSA was placed in an aluminum pan and heated from 25 to 650 °C under nitrogen atmosphere at a rate of 10 °C/min.

The differential scanning calorimetry (DSC) of four WPU-PSAs was characterized by TA Instruments DSC Q200 (TA Instruments, New Castle, DE, USA). To eliminate the thermal history the samples were heated at 10 °C/min from 25 to 200 °C, cooled to −70 °C at 10 °C/min and heated again to 200 °C at 5 °C/min to obtain the glass transition temperature (Tg).

Swelling tests were performed for the four WPU-PSAs. As such, 2 g of each WPU-PSA were spread in a glass dish and placed in an oven at 65 °C for 12 h. The dried films were immersed in a THF solution at room temperature and the dissolution was observed after 24 h.

Soxhlet extraction was utilized to test gel content and liquid absorption of films of PSA2 and PSA3. A film (approx. 2 g) was wrapped in nylon gauze with the constant weight of W_1_, and then the wrapped film was placed at 70 °C for standing for 12 h before weighing (weight W_2_). Then, the wrapped film was extracted in tetrahydrofuran at 90 °C in a Soxhlet extractor for 24 h. The wrapped film after Soxhlet extraction was weighed and recorded as W_3_. To remove the absorbed solvent the wrapped film was dried at 60 °C for 12 h, and then the weight was recorded as W_4_. The gel content and swelling rate were calculated according to equations 1 and 2, respectively.

Gel content (wt%) = (W_4_ − W_1_)/(W_2_ − W_1_) × 100% (1)

Swelling rate (wt%) = (W_3_ − W_1_)/(W_2_ − W_1_) × 100% (2)

The average molecular weights of the soluble portion of WPU-PSAs were determined by gel permeation chromatography (GPC, Waters Corporation, Milford, CT, USA) at the column temperature of 40 °C in tetrahydrofuran (THF) as an eluent using polystyrene standards. A dried film of approximately 5 mg was dissolved in 5 mL of THF. The polymer-containing THF solvents were filtered before it could be (prior to analysis) injected into the instrument, at least 150 µL of solutions for molecular weight determination.

The loop tack of WPU-PSAs was measured at 25 and 60 °C by KINSGEO^®^ KJ-603 (Guangdong Kejian Instruments Co., Dongguan, China), and the tester crosshead speed was set at the rate of 100 mm/min. Polyethylene terephthalate (PET) film backing 0.05 mm in thickness was used for test preparation (length 150 mm and width 25 mm). The WPU sample was uniformly spread on the PET film backing using a cube applicator, then dried at 70 °C for 20 min. The dry adhesive layer of WPU-PSAs covering the PET film backing was approximately 40–50 μm in thickness (Figure S1). The weight of the test strip itself being applied as the only force; each strip article was brought into contact with the surface of stainless steel 304 plate. The contact area was of 25 by 25 mm. The maximum force to separate the strip from the plate was recorded to evaluate the loop tack, as well as the mode of failure (adhesion, cohesion or transfer).

The holding time of WPU-PSAs was characterized by PARAM^®^CZY-GS (Jinan Labthink Electromechanical Technology Co., Jinan, China). The preparation method of the test strips was similar to that of loop tack, except that test strips here were 50 mm long and 25 mm wide. The strip was bonded to the stainless steel 304 plates smoothly, and the contact area of each steel plate is 25 by 25 mm. The assembled construction was rolled back and forth once with a 2000 ± 40 g rubber roller. Each bonded construction was hung with a weight of 1000 ± 40 g loaded. Subsequently, the bonding adhesive layer crept under the action of external force parallel to the bonding surface. When a construction was separated, the corresponding timing stopped automatically. The test values were the time in minutes to separation (Figure S2).

The 180° peel strength of WPU-PSAs were characterized in a SUNDOO^®^ SPJ universal tester and the separation speed was set at 150 mm/min. The preparation method of the test (Wenzhou Bino Instrument Co., Wenzhou, China) strips was similar to that of loop tack, except that test strips here were 300 mm long and 25 mm wide (Figure S3). A stainless steel 304 plate of dimensions 150 × 50 × 1 mm was used. The assembled construction was rolled back and forth once with a 2000 ± 40 g rubber roller. A length of 150 mm of the joint was peeled, and the values of initial 60 mm-length at one end were discarded. The average force to peel the strip from the stainless steel 304 plate was recorded to evaluate the 180° peel strength. At least three strips were tested.

The viscoelasticity of WPU-PSAs films was evaluated by temperature sweep and frequency sweep experiments in a Discovery HR-2 hybrid rheometer (TA Instruments, New Castle, DE, USA), and plate-plate geometry (25 mm parallel plate, ETC aluminum disposable type) was used. Temperature sweep experiments were carried out at a frequency of 1 Hz and strain amplitude of 2.5%, and the temperature range was from 25 to 105 °C. Oscillatory frequency sweep experiments were performed at 25 °C with an angular frequency range from 0.1 to 100 rad/s.

## 4. Results and Discussion

### 4.1. Structural Characterization of the WPU-PSAs

Four WPU-PSAs were obtained with four kinds of chain extenders listed in Table 1. For the PSA1, the isocyanate-terminated prepolymer molecules could only react with the hydroxyl group, and carbamate groups were formed, while for the other three kinds of WPU-PSAs, the isocyanate-terminated prepolymer molecules could react with the hydroxyl and amino groups, and then carbamate and urea groups were formed. Furthermore, the different reactivity of -OH, -NH_2_ and -NH- with isocyanate group and the influence of the steric hindrance of benzene ring led to different gel contents and corresponded molecular weight distributions in the four WPU-PSAs.

The ATR-FTIR spectra of the four WPU-PSAs are presented in Figure S4. It was found that the four WPU-PSAs have similar IR spectra, and the difference of chain extender components is not obvious in the IR spectra. The broad N-H stretching of urethane group is at peak of 3329 cm^−1^, the C-H stretching band of -CH_3_ and -CH_2_ group at 2975–2830 cm^−1^, the C=O stretching of urethane and urea group at 1730 cm^−1^, the N-H stretching of amide group at 1534 cm^−1^ and the C-O-C bands of ether group at 1104 cm^−1^.

The thermal weight loss and the thermal weight loss rate of the four WPU-PSAs were characterized by TGA and derivative TGA (DTGA), respectively (Figure S5). As the polyol contents of the four WPU-PSAs were the same, the temperature onset (T_onset_) of thermal decomposition were similar. The temperature of mass loss of 5% (T_5%_) and 50% (T_50%_) are listed in Table S1. PSA2 and PSA3 have cross-linked structure, so they have better thermal stability. The temperature T_50%_ of PSA2 and PSA3 was higher than that of both PSA1 and PSA4. The DTGA of all WPU-PSAs was shown in Figure S5b, where 326–354 °C and 385–410 °C represented the decomposition temperature range of hard and soft segments of WPU-PSAs, respectively. Due to the high soft segment content of the four samples, the main heat loss was concentrated at 385–410 °C.

As shown in Figure S6, each DSC thermogram of the four WPU-PSAs presents one Tg at the range of −52.24 to −51.58 °C due to the same soft segments. Whereas, the glass transition temperatures corresponding to the hard segments in the WPU are not observed in the high temperature region [13], probably due to the low content of hard segments of 26.5–27.3%.

**Figure 3:**
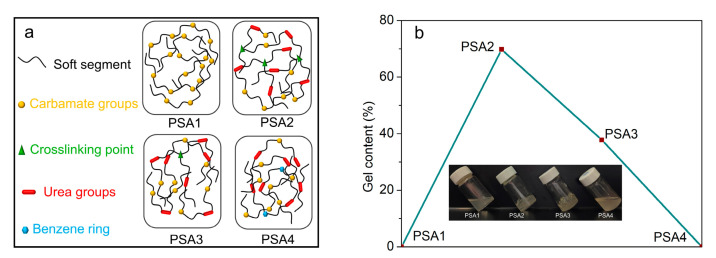
(**a**) Schematic diagram of the structure of the four WPU-PSAs. (**b**) The swelling condition of WPU-PSAs in THF and the corresponded gel contents. PSA1 and PSA4 dissolved completely, and PSA2 and PSA3 swollen with the gel content of 70% and 38%, respectively.

The use of different chain extenders led to the WPU-PSAs with different macromolecular structures. All four polymers contain the same soft segments but different hard segments, as illustrated in Figure 3a. The architecture of the polymer was determined by the measurement of swelling and gel content. Dried films of the four WPU-PSAs were immersed in THF for 6 h. It was found that PSA2 and PSA3 were swollen (not completely dissolved), while PSA1 and PSA4 were completely dissolved, as shown in Figure 3b. What is more, PSA2 and PSA3 had a swelling rate in THF of 1344% and 879%, respectively. Thus, PSA2 and PSA3 could be considered containing a fraction of three-dimensional network structure, while PSA1 and PSA4 were of linear structure. The cross-linked part and the sol part constructed a broad molecular weight distribution together.

Soxhlet extraction was used to determine the gel content and swelling rate of both PSA2 and PSA3, and the result was listed in Table 2. PSA2 with the highest gel content of 70% was mainly cross-linked, while PSA3 with a higher gel content of 38% was mainly linear. This should be due to the different content of primary amine groups used in the preparation of PSA2 and PSA3. The crosslinking points illustrated in Figure 3a are concluded the combinations of carboxyl acid group and primary/secondary amine group. The further evidences are presented in the Supplementary Material. Recent works disclosed the strong interaction between the -COOH and -NH- or -NH_2_ in ethanol at room temperature [29], and even the direct coupling formation of amide bond at room temperature from –COOH and -NH_2_ on the graphene oxide surface [30] or on the Au (111) surface [31].

GPC was performed in the determination of the sol molecular weight of the four WPU-PSAs (Figure S7), while the molecular weights of the cross-linked parts of PSA2 and PSA3 could not be obtained by GPC [32,33]. Molecular weight parameters of the soluble portion of the four WPU-PSAs was listed in Table 2, including the number-average molecular weight, weight-average molecular weight, polydispersity index (PDI) and standard deviation of slice log M_w_. There was no significant difference in molecular weight and PDI values of the soluble part of the four samples. The results suggested that the difference of the adhesive performances of the four WPU-PSAs might not mainly be determined by the length of the macromolecular chains, but by other factors, such as the gel content and the nature of the soluble macromolecular chains.

### 4.2. Adhesive Performances of the WPU-PSAs

The loop tack, holding time and 180° peel force of the four WPU-PSA samples at 25 and 60 °C were tested. The results were shown in Table 3.

**Figure 4:**
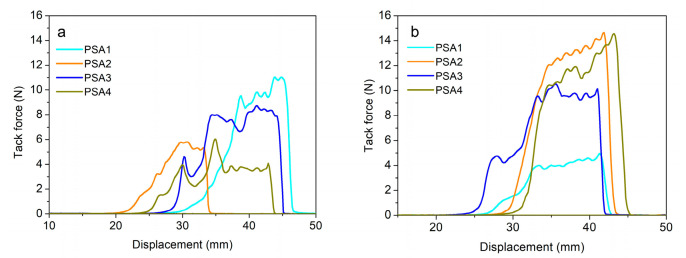
WPU-PSAs loop tack at 25 °C (**a**) and 60 °C (**b**).

At 25 °C, pressure sensitive adhesive performances of PSA1 were quite different from PSA2, PSA3 and PSA4 in Figure 4a. PSA1 corresponded to higher loop tack, but the other three WPU-PSAs had better shear resistance (longer holding time under a specific shear force) and higher 180° peel forces. The higher loop tack of PSA1 should derive from its better fluidity and reasonable interaction between the adhesive molecules and the surface layer of the adherent at 25 °C [2]. Since all chain extenders of PSA1 were BDO rather than a mixture of BDO with an amine, PSA1 had no urea groups, and therefore, had weaker interaction between the polymer chains than the other three WPU-PSAs. What is more, the GPC molecular weight of PSA1 was just similar to those of the other three. Considering the above two factors, there should exist less entanglement of polymer chains and smaller obstacles of polymer chains motions in PSA1, which made it better fluidity and lower cohesion at 25 °C. In fact, better tack was usually associated with lower cohesion, with worse shear resistance strength and peel strength, which was generally the case. However, the cohesion of PSA could not be too small, as some degree of cohesion was necessary to maintain some degree of integrity of the adhesive joint. As shown in Figure 4b, PSA1 had better fluidity and better tack at 25 °C, but with the increase of temperature to 60 °C, its fluidity gradually approached that of a liquid, and no longer had the necessary cohesion as an adhesive, thus had worse tack at 60 °C. On the contrary to PSA1, the other three WPU-PSAs had lower fluidity and lower tack at 25 °C. When the temperature increased, the fluidity of the three adhesives increased and their contact with the same adherent was better. Therefore, the tack of the three adhesives at 60 °C was better than that at 25 °C. Nevertheless, better liquidity also meant the bulk part of PSA bonding joint was less resistant to external force, so as to have lower shear-resistance strength and shorter holding time in the creep test. Thus, the balance between the cohesion and fluidity of PSA was a very important factor affecting the pressure sensitive adhesion properties.

With the temperature increasing from 25 to 60 °C, the holding time of all WPU-PSAs decreased significantly except PSA2. As showed in Table 3. PSA2 presented a holding time not less than 100 h both at 25 and 60 °C, which suggested that the temperature change hardly affected its shear resistance strength in this test situation and PSA2 should have the best thermal stability among the four WPU-PSAs at 25–60 °C. The molecular structure of PSA2 contained 30 wt% of linear chains and 70 wt% of cross-linked networks, as shown in Table 2. With the increase of temperature, the tack of PSA2 increased the most and the holding time changed the least, which should be attributed to the balance of content between linear structures and cross-linked networks of the polymer, and the strategy could be used in the rational design of WPU-PSAs.

**Figure 5:**
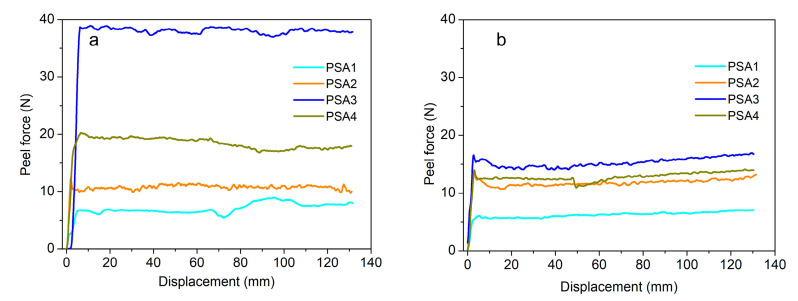
The peel force of WPU-PSAs at 25 °C (**a**) and 60 °C (**b**).

The 180° peel forces of the four WPU-PSAs were tested at 25 and 60 °C, and the results were listed in Table 3 and Figure 5. With the increase of temperature, the 180° peel forces of three WPU-PSAs decreased to different degrees, except PSA2. PSA2 had the best thermal stability in the test of 180° peel strength of the four WPU-PSAs, just as it performed the best in the test of holding time. As expected, PSA1 had the smallest value of 180° peel force. Unexpected, with the increase of temperature from 25 to 60 °C, the decrease of 180° peel strength value of PSA3 was greater than that of PSA4, which was not consistent with their performance in the test of holding time. Conversely, with the increase of temperature, the decrease of holding time of PSA4 was greater than that of PSA3. It seemed that the different stresses used in the two kinds of tests caused this result. The test of 180° peel strength was carried out at a much higher strain rate than the test of holding time, and the stress corresponding to the former was larger. Compared with PSA3, the polymer chains of PSA4 had more deformation lag under the external strain of higher rate. In other words, PSA4 showed more entanglement than PSA3 under a larger stress, such as peel force, while PSA4 showed less cohesion than PSA3 under a smaller stress, such as creep force [34,35]. PSA4 contained a higher content of urea groups and phenyl groups, so its polymer chains lagged strongly. Therefore, with the temperature increased from 25 to 60 °C, the attenuation of 180° peel strength of PSA4 was weaker than that of PSA3. In combination with the result of PSA2, it revealed that both the cross-linked structures and linear structures with strong macromolecular interactions could affect the 180° peel strength of PSA, and the strategy could be used in the rational design of PSA.

**Figure 6:**
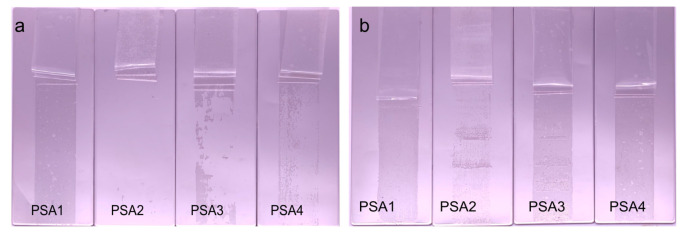
The residuals of WPU-PSAs at 25 °C (**a**) and 60 °C (**b**).

The residual glue amount of the bonding constructions after 180° peel test was also a key indicator to evaluate the performance of WPU-PSAs. The condition of the residual glue amount of four WPU-PSAs at 25 and 60 °C was shown in Figure 6. PSA2, PSA3 and PSA4 had less residual glue than PSA1 at 25 °C. Obviously, residues of all WPU-PSAs at 60 °C increased to varying degrees than at 25 °C. The amount of residual glue of PSA2 increased with the increase of temperature, but its degree of increase was much less than that of the other three. Residues of PSA1 were the most obvious both at 25 and 60 °C. Since 60 °C was higher than the flow points, residues of both PSA3 and PSA4 also increased significantly from 25 to 60 °C. This revealed that the residual glue of WPU-PSAs could be controlled by adjusting the crosslinking degree and the interaction forces between macromolecules. If there is little or no residual glue, we have the possibility to reuse PSA. The problems of heat resistance and adhesive residue might be solved by improving the predesigned working temperature of WPU-PSAs.

The above results presented the different natures of four chain extenders and their effects on the syntheses of the four WPU-PSAs. Neither PSA1 nor PSA4 had detectable gel, while both PSA2 and PSA3 had obvious gel. PSA1 and PSA4 were of similar molecular weight and molecular weight distribution, but they had different adhesive performances. This should be caused by the fact that PSA4 contained more urea bonds and phenyl groups than PSA1. In addition, due to the action of benzene ring, the reaction activity of the amino group in ODA was not as good as that in AEEA and EDA, so there was no such cross-linking structure in PSA4 as in PSA2 and PSA3. What is more, PSA2 had higher gel content of 70% than PSA3 of 38%, because EDA used in PSA2 had more highly active amino groups than AEEA used in PSA3. EDA and AEEA could be used as a high-functionality cross-linker to control the pressure sensitive adhesion properties of WPU-PSAs. It indicated that both the content of cross-linked structures and linear chains with strong macromolecular interactions in a polymer could significantly affect the adhesive performances of WPU-PSAs. WPU-PSAs with good tack at 25 or 60 °C could be reasonably designed and obtained by using chain extenders BDO, EDA, AEEA and ODA in this preparation progress.

### 4.3. Rheological and Viscoelastic Properties of the WPU-PSAs

As shown in the previous discussion, except PSA being too fluid to maintain the necessary integrity as an adhesive, better liquidity generally means higher loop tack and, also, better pressure sensitive performance. On the other hand, lower loop tack and longer holding time generally indicate the harder mobility of polymer chains. The fluidity of polymer chains corresponds to its dynamic modulus. In order to link the macroscopic adhesive performances with the microscopic macromolecular motion, the relationship between the adhesive performances and polymer molecular structures was analyzed from the perspective of rheology. The stress-strain response of WPU-PSAs could be obtained by rheological analysis, and frequency sweeps and temperature sweeps were conducted in the linear viscoelastic region (LVE).

**Figure 7:**
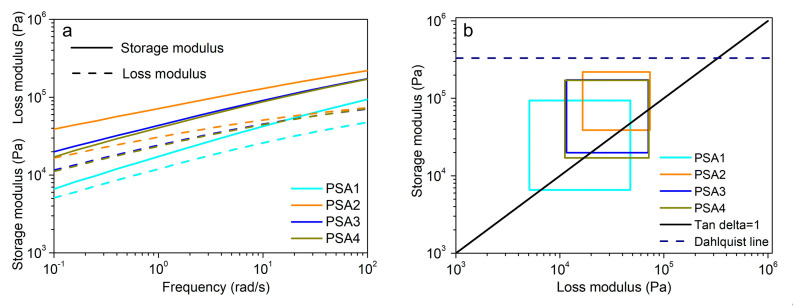
(**a**) Frequency sweeps of the four WPU-PSAs in the linear viscoelastic interval with an angular frequency range of 0.1 to 100 rad/s at 25 °C. (**b**) Like Chan G′s viscoelastic window of PSA1, PSA2, PSA3 and PSA4 at 25 °C. The dash line represents the Dalquist criterion line. The black solid line indicates G′ = G″ (tan delta = 1).

Frequency sweeps of the four WPU-PSAs were carried out with an angular frequency range from 0.1 to 100 rad/s. As shown in Figure 7a, with the increase of frequency, both the storage modulus (G′) and loss modulus (G″) increase. In the frequency interval, the G′ of PSA1 is the lowest, and the G′ of PSA2 is the highest. The G′ curves of PSA3 and PSA4 are very similar, and they lie between the G′ curves of PSA1 and PSA2. The like ChanG′s viscoelastic window in Figure 7b is obtained from the values of G′ and G″ corresponding to frequencies of 0.1 and 100 rad/s [8], and can be used to predict whether the WPU-PSAs will form an effective contact at a given temperature. The G′ of all the WPU-PSAs is in line with the Dahlquist criterion (G′ < 3.3 × 10^5^ Pa) at 25 °C. In other words, all the four WPU-PSAs are pressure sensitive at 25 °C, and the viscoelastic area of all samples are situated in the center area of the like ChanG′s viscoelastic window, which are general PSA at room temperature [5]. Furthermore, PSA1 is prone to situate in the lower left quadrant, indicating PSA1 trended to be a removable WPU-PSAs, while PSA2 is prone to situate in the higher right quadrant, indicating PSA2 trended to be a heat-resistance WPU-PSAs.

Unlike the obvious relationship between the G′ and the adhesive performances, such as the Dalquist criterion line which is closely related to tack, the influence of G″ is not obvious and less concerned on. As shown in Figure 7a, the G″ of PSA4 is almost equal to that of PSA2 at the frequency of 100 rad/s, which means the G″ of PSA4 increases by more than that of PSA2 as the frequency increases from 0.1 to 100 rad/s. The frequency of 100 rad/s corresponds approximately to the frequency of peel operation [8]. Correspondingly, at the strain rate of 180° peel, PSA4 could show good resistance to external forces or temperature changes. Although the average molecular weight and PDI of PSA4 are similar to those of the soluble part of PSA2, there exist more linear macromolecular chains with strong molecular interactions in PSA4 than in PSA2, which could cause more entanglements at a high strain rate. Therefore, at 100 rad/s, the motion lag of polymer chains of PSA4 results in its G″ close to that of PSA2. As listed in Table 3, when the temperature increased from 25 to 60 °C, PSA4 has a less decline of peel performance than PSA3, while a greater decline of holding time performance. The motion lag of polymer chains has a greater effect on the 180° peel strength than on the holding time performance for PSA4. It appears that the G″ of PSA4 is more important for its 180° peel strength than for its holding time performance.

As discussed above, both elasticity (G′) and viscosity (G″) plays an important role on the tack, holding time and 180° peel performance of PSA, which can be controlled by adjusting the content of linear structures with strong interaction, and the gel content.

**Figure 8:**
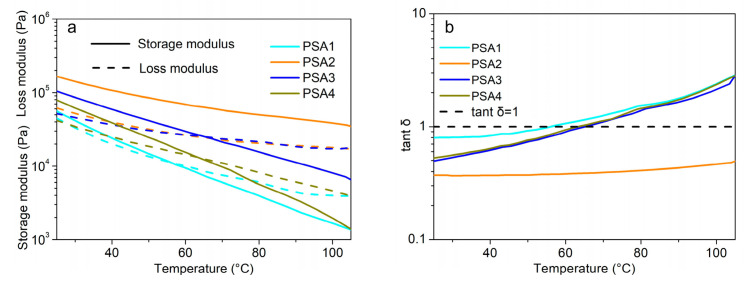
(**a**) Temperature sweeps of the four WPU-PSAs from 25 to 105 °C. (**b**) Loss factor of four WPU-PSAs over a temperature range from 25 to 105 °C.

Temperature sweeps were carried out at a frequency of 1 Hz and a strain of 2.5%. As shown in Figure 8a, with the increase of temperature, both the G′ and G″ of the four WPU-PSAs decrease. In the temperature interval of 25 to 105 °C, the G′ of PSA1 is the lowest, and the G′ of PSA2 is the highest, indicating that the fluidity of PSA1 is the best and that of PSA2 is the worst in this temperature range. Thus, PSA1 has better wetting and adhesion to the adhered material, and better loop tack at 25 °C. However, as the temperature rises to 60 °C, the G′ of PSA1 becomes too small and result poor loop tack of PSA1 at 60 °C. The G′ of PSA2 at 60 °C is very close to that of PSA1 at 25 °C, which is a clue that PSA2 may provide a good tack at 60 °C just well as PSA1 at 25 °C. As the temperature increase to 100 °C, the G′ of PSA1 is almost equal to that of PSA4, but the G′ of both PSA2 and PSA3 are still much greater than that of PSA1 and PSA4. As the temperature increases, the decrease rate of G′ of both PSA2 and PSA3 are slower, which is attributed to their gel content and the corresponded thermal stability. It is found that the G′ variation of WPU-PSAs with the temperature and the corresponded adhesive performances are affected greatly by the gel content of the polymer. It can be seen that the G′ curves of the four WPU-PSAs can provide their information of adhesive performances at higher temperatures.

The loss factor is the proportion of G″ to G′ and the temperature when the loss factor is 1 is called flow point temperature (T_f_). As shown in Figure 8b, the T_f_ of PSA1 is 55 °C, and those of PSA3 and PSA4 were 67 °C and 65 °C, respectively. The flow point temperatures of WPU-PSAs are consistent with their amount of residual glue at 60 °C. Residues of both PSA3 and PSA4 increased significantly from 25 to 60 °C. Throughout the temperature scanning interval, PSA2 displays no flow point temperature. Over the temperature of T_f_, PSA1 is dominantly viscous and too fluid to act as an adhesive. As a result, PSA1 lacks the necessary cohesion required by an adhesive and shows poor loop tack at 60 °C. On the contrary, T_f_ of PSA2 is much higher, and its maximum loss factor is only 0.5 (Figure 8b) which means the G′ is at least twice the G″. At the temperature range of 25 to 105 °C, PSA2 is dominantly elastic, being attributed to the high content of gel. Test results in Table 3 showed that with the increase of temperature from 25 to 60 °C, the holding time of PSA2 retained, while the holding time of the other three WPU-PSAs decreased sharply. The different holding time performances of the four WPU-PSAs at 60 °C were in accordance with their different T_f_.

**Figure 9:**
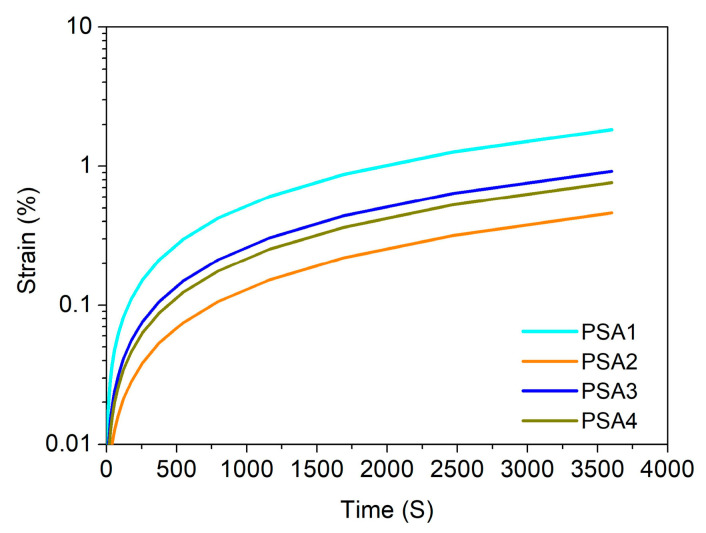
Creep curves of four WPU-PSAs at 25 °C under stress of 1 Pa.

A frequency sweep spectrum can be converted into a creep spectrum. As shown in Figure 9, the creep spectrum of each WPU-PSAs is significant different at 25 °C. Under the same stress in a certain range, the strain of PSA2 is the smallest, that of PSA1 is the greatest, and that of both PSA3 and PSA4 is between that of PSA1 and PSA2. Furthermore, strain of PSA1 is nearly seven times that of PSA2 at the time of 3.6 × 10^3^ s. On the other hand, it is also shown that PSA1 needs the shortest time to reach a deformation value less than 1%. There is a correlation between the creep performances and the holding time performance. For example, the creep spectra of PSA3 and PSA4 exhibit only slight difference, and likewise, their holding time performances are similar (Table 3). The creep results are in good agreement with the adhesive performances test results.

## 5. Conclusions

Four WPU-PSAs were prepared by using BDO, BDO + EDA, BDO + AEEA and BDO + ODA as chain extender, respectively, and their structure characterization, adhesive performances change with temperature, and rheological behaviors were investigated.

The WPU-PSA made with BDO + EDA contained the highest gel of 70% because of the highest number of aliphatic -NH_2_ group in chain extender. That made with BDO + AEEA contained 38%. The other two contained no gel. Number-average molecular weight of each sol of the four WPU-PSAs in THF ranged between 41,097 and 43,544 g/mol, with their PDI ranged from 1.33 to 1.38. The four WPU-PSAs had similar T_g_ (at range of −52.24 to −51.58 °C), due to the same soft segment, while they had obviously different T_f_ (at 55 °C, over 105 °C, 67 °C and 65 °C, respectively), due to the different hard segment.

The adhesive performances of the WPU-PSAs varied with temperature. For tack, the WPU-PSAs increased significantly with the increase of temperature from 25 and 60 °C, except the one made with BDO decreased significantly. The one made with BDO showed the highest tack at 25 °C and the lowest tack at 60 °C, while the one made with BDO + EDA showed the lowest tack at 25 °C and the highest tack at 60 °C. For holding time, the WPU-PSAs decreased drastically with the increase of temperature from 25 and 60 °C, except the one made with BDO + EDA remained the same value within 100 h. For 180° peel force, the WPU-PSAs all decreased to varying degrees with the increase of temperature from 25 and 60 °C.

The adhesive performances of the WPU-PSAs varied with temperature were ascribed to the amount of gel in the structures of the four WPU-PSAs and the interactions between polymer chains. The gel content played important role for the term of holding time, and the WPU-PSA of the highest gel content presented the longest holding time at both 25 °C and 60 °C. Both a right amount of gel and a strong interaction between macromolecular chains were important for the term of 180° peel force. In addition, the strong interaction between macromolecular chains could do the same thing as the gel in affecting adhesive performances and T_f_.

The adhesive performances of the WPU-PSAs varied with temperature could be correlated to the rheological behaviors well. All the four WPU-PSAs presented suitable G′ value at both 25 °C and 60 °C, which meant they were PSA at both 25 °C and 60 °C. Furthermore, adhesive performances of the WPU-PSAs were also correlated with the gap between their T_f_ and bonding temperature (working temperature). The smaller the gap took better tack performance for each of the WPU-PSAs. The smaller the gap took worse holding time performance for each of the WPU-PSAs except the one made from BDO + EDA within 100 h (this time too short to show its deterioration). The gap affected the 180°peel force relatively complicatedly, as the 180° peel tested at a higher rate than both tack and creep.

By using plots of the dynamic moduli versus temperature, the G′ value around the bonding temperature and the gap between the T_f_ and the bonding temperature (working temperature), adhesive performances at a certain temperature could be inferred and adhesion failures at a too high or too low temperature could be analyzed reasonably or be used positively. Thus, the relationship among macromolecular structure, adhesive performances change at different working temperatures and rheological behaviors could provide a new perspective point to understand and adjust the properties of WPU, and help reducing costly trial and error. The study here may provide mechanistic insights about the design of WPU-PSAs and other kinds of adhesives with designated working temperature, although other kinds of adhesives require additional technical means to provide specific properties that pressure sensitive adhesives do not have.

## Figures and Tables

**Table 1:** Composition for the syntheses of WPU-PSAs.

Sample Code	Composition (mol.)							
	PPG	HHTPB	HDI	DMPA	BDO	EDA	AEEA	ODA
PSA1	0.9	0.1	3	1	1	0	0	0
PSA2	0.9	0.1	3	1	0.8	0.2	0	0
PSA3	0.9	0.1	3	1	0.8	0	0.2	0
PSA4	0.9	0.1	3	1	0.8	0	0	0.2

**Table 2:** The gel content, swelling rate, number-average molecular weight (${\bar{M}}_{n}$) weight-average molecular weight (${\bar{M}}_{w}$) and PDI of the sol of the four WPUs.

Sample Code	Gel Content (%)	Swelling rate (%)	${\bar{M}}_{n}(g/\mathrm{mol})$	${\bar{M}}_{w}(g/\mathrm{mol})$	PDI $\left({\bar{M}}_{n}/{\bar{M}}_{w}\right)$	Standard Deviation of Slice Log M_w_
**PSA1**	0	0	41,097	54,869	1.33	0.23
**PSA2**	70	1344	42,494	58,666	1.38	0.25
**PSA3**	38	879	42,697	59,406	1.39	0.25
**PSA4**	0	0	43,544	61,162	1.38	0.25

**Table 3:** Adhesive performances of four WPU-PSAs at 25 and 60 °C, respectively.

Sample Code	Loop Tack (N/25 mm)		Holding Time (h)		180° Peel Force (N/25 mm)	
	25 °C	60 °C	25 °C	60 °C	25 °C	60 °C
PSA1	11.0	5.0	0.4	0.1	7.2	6.4
PSA2	5.8	14.7	100.0	100.0	10.7	11.4
PSA3	8.7	10.5	6.2	1.0	36.9	15.3
PSA4	6.1	14.6	9.1	1.1	20.3	12.7

## Data Availability

Not applicable.

## Supplementary Materials

The following supporting information can be downloaded at: https://www.mdpi.com/article/10.3390/ma15062011/s1, Figure S1. Side view and top view of loop tack test (left). Specimen loop fixture and stainless steel 304 plate (right); Figure S2. Holding time test; Figure S3. 180° peel force test; Figure S4. ATR-FTIR spectra of the four WPU-PSAs; Figure S5. The thermal gravimetric analysis (TGA) (a) and derivative TGA (DTGA) (b) curves of the four WPU-PSAs; Figure S6. DSC thermograms of the four WPU-PSAs; Figure S7. Molecular weight distribution of the four WPU-PSAs sols; Figure S8. Schematic diagram of the combination of -COOH and -NH_2_; Figure S9. (a) Infrared spectroscopy of the reactants (EDA, DMPA) and product (DMPA-EDA) in methyl ethyl ketone. (b) Infrared spectroscopy of the reactants (DMPA, TEA, EDA) and product (DMPA-TEA-EDA) in methyl ethyl ketone. (c) and (d) are partial Infrared spectroscopy analysis of (a) and (b), respectively; Table S1: Solid contents, glass transition temperature (Tg), temperature onset (T _onset_) of thermal decomposition, and temperature of 5% (T_5%_) and 50% (T_50%_) mass loss, respectively. Refs. [29,30,31,36] are cited in supplementary materials.
