# Supplementary material for: Rational Design of Waterborne Polyurethane Pressure Sensitive Adhesives for Different Working Temperatures
Source: Materials (Basel). 2022 Mar 8;15(6):2011. doi: 10.3390/ma15062011 (PMC8949434; doi:10.3390/ma15062011)
Supplement: Supplementary file 1 [file materials-15-02011-s001.zip › materials-1584203-supplementary-2.pdf]

materials

Article

Rational Design of Waterborne Polyurethane Pressure Sensitive Adhesives for Different Working Temperatures

Hui Zhao [1,2], Ying Xu [2,*], Zhen Luo [2], Cui-Ran Gong [2], Yang-Qing Zheng [2] and Li-Ming Yu [2]

1 College of Chemistry & Materials Science, Fujian Normal University, Fuzhou 350007, China; huizhao@fjirsm.ac.cn
2 Key Laboratory of Coal to Ethylene Glycol and Its Related Technology, Fujian Institute of Research on the Structure of Matter, Chinese Academy of Sciences, Fuzhou 350000, China; zhenluo@fjirsm.ac.cn (Z.L.); gongcuiran@163.com (C.-R.G.); yqzheng@fjirsm.ac.cn (Y.-Q.Z.); yuliming@fjirsm.ac.cn (L.-M.Y.)
* Correspondence: westxu@fjirsm.ac.cn

Abstract: The appropriate pressure sensitive adhesion performances at working temperature are vital for the applications of waterborne polyurethane (WPU). Understanding the relationship among rheological behaviors, macromolecular structures and adhesive performances can be very useful to the rational design of waterborne polyurethane pressure sensitive adhesives (WPU-PSAs) for different operating temperatures, as well as other kinds of adhesives. In this study, four kinds of WPU-PSAs were prepared by reacting polypropylene glycol (PPG), hydrogenated hydroxyl-terminated polybutadiene (HHTPB), dimethyl alcohol propionic acid (DMPA), 1,6-hexamethylene diisocyanate (HDI) and four kinds of chain extenders. Gel permeation chromatography (GPC), swelling and rheology tests were used in parallel with an analysis of adhesive performances of the dried films of the adhesives. Results showed that, in addition to the nature of chain extenders playing a role on the rheological behaviors and adhesive performances of polymer, the gel content could be used to adjust the macromolecular structure and molecular weight distribution of polymer, thus distinctly affected the adhesive performances of PSA. The relationship among rheological behaviors, macromolecular structure and adhesive performances was investigated, and the rational design of WPU was achieved with appropriate pressure sensitive adhesion properties for different working temperatures of 25 and 60 °C.

Keywords: waterborne polyurethane (WPU); pressure sensitive adhesive (PSA); rheology; adhesive performance; rational design.

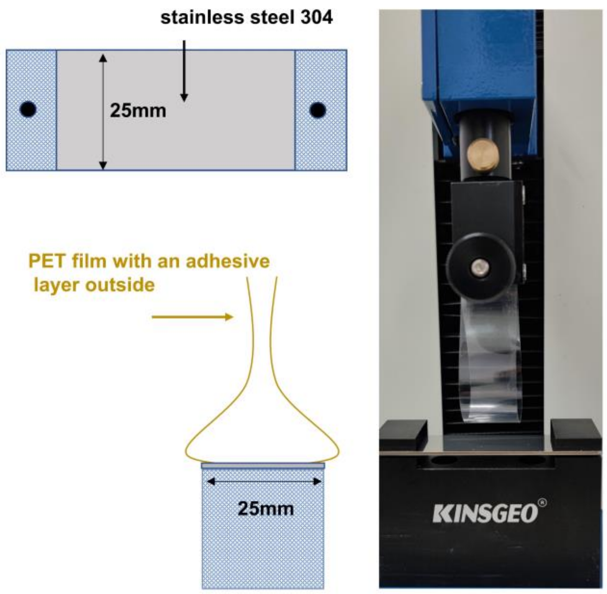

Figure S1. Side view and top view of loop tack test (left). Specimen loop fixture and stainless steel 304 plate (right).

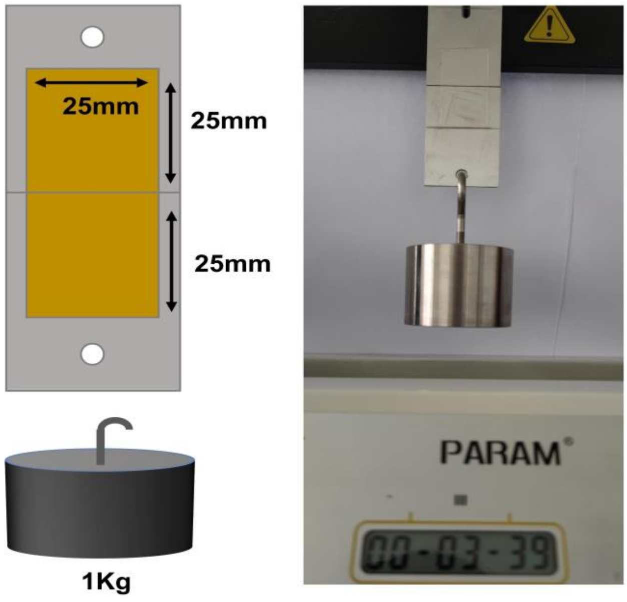

Figure S2. Holding time test.

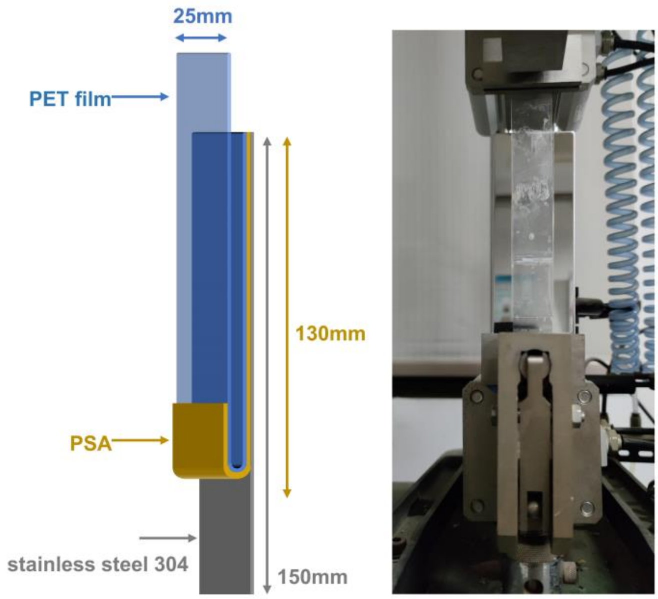

Figure S3. 180° peel force test.

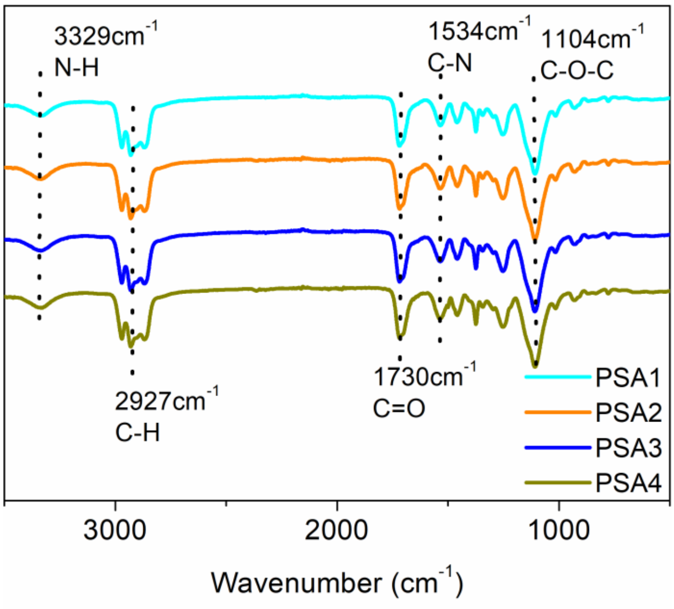

Figure S4. Fourier transformed infrared spectroscopy (ATR-FTIR) spectra of the four waterborne polyurethane pressure sensitive adhesives (WPU-PSAs).

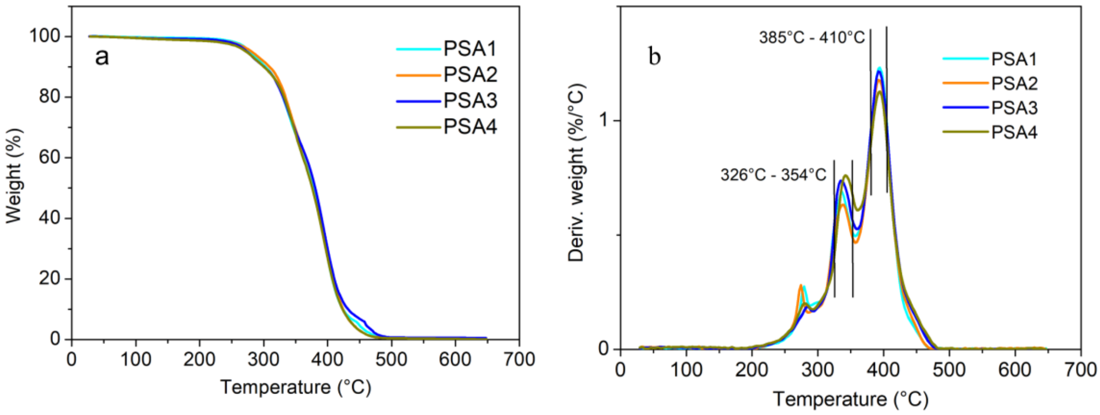

Figure S5. The thermal gravimetric analysis (TGA) (**a**) and derivative TGA (DTGA) (**b**) curves of the four WPU-PSAs.

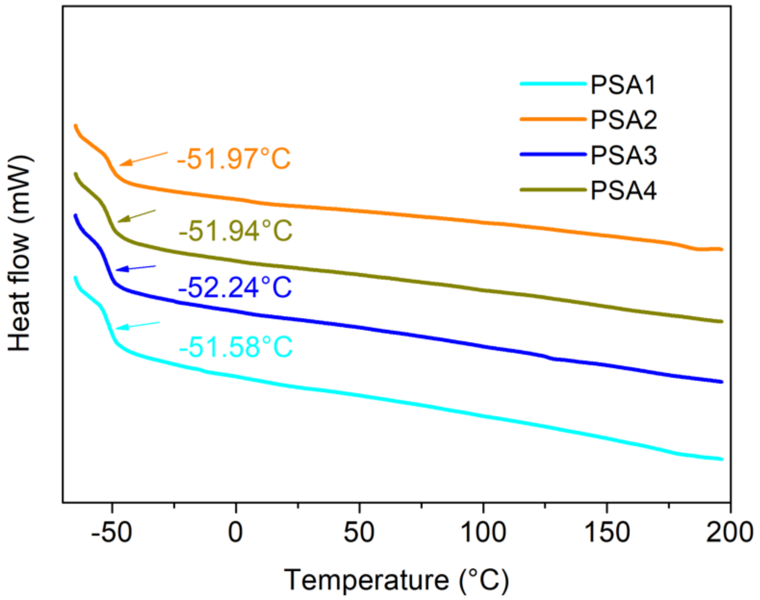

Figure S6. The differential scanning calorimetry (DSC) thermograms of the four WPU-PSAs.

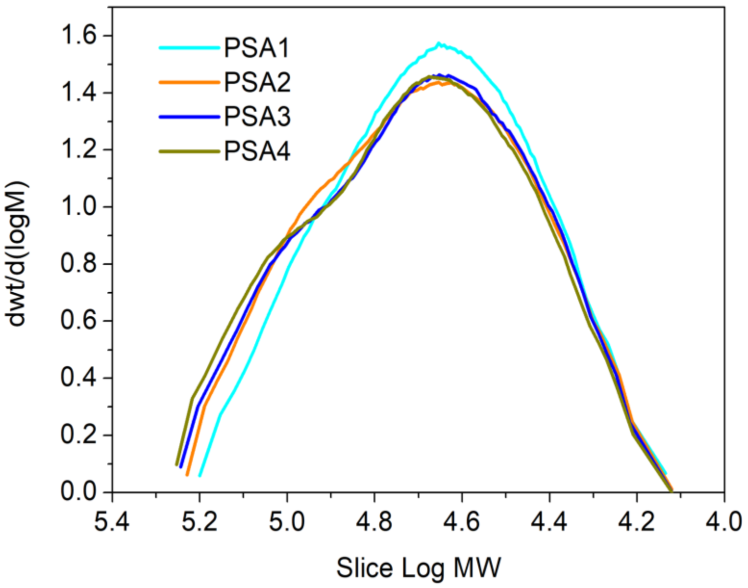

Figure S7. Molecular weight distribution of the four WPU-PSAs sols.

The evidences for "the crosslinking points illustrated in Figure 3(a) are concluded the combinations of carboxyl acid group and primary/secondary amine group"

The crosslinking network of PSA2 and PSA3 may be due to the interaction between the primary/secondary amine of EDA/AEEA and the carboxylic acid (COOH) of DMPA, the combination -COOH·NH_2- (or -COOH·NH-), as shown in Figure S8 [1] Three aspects will be explained and illustrated. The following triethylamine and 2,2′-bis(hydroxymethyl)propionic acid are denoted by TEA and DMPA, respectively.

1) Using a similar preparation of PSA2, three waterborne polyurethane dispersions were prepared with three TEA-DMPA ratios. And the three TEA-DMPA ratios were used to control the neutralization degree of –COOH of DMPA in three levels of percentage (110%, 100% or 80%, respectively), and the impact on gel was determined.

The Soxhlet extraction experiments in tetrahydrofuran (THF) showed that there were lots of gel in the three samples. The results showed that the interactions between the chain extender and -COOH groups was still be possible, although the chain extension was carried out after neutralization of the proton of DMPA.

When the proton of DMPA was neutralization by TEA, the interaction between -NH_2 of the chain extender and -COOH group was presumed to be due to the formation of the combinations -COOH·NH_2, as the stronger base of -NH_2 group than $N(CH_2CH_3)_3$. This was illustrated in Figure S8.

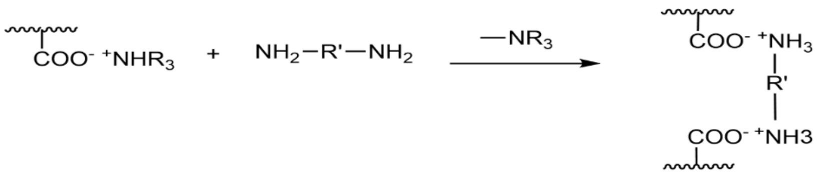

Figure S8. Schematic diagram of the combination of –COOH and -NH_2.

2) Comparative experiments were carried out to discern the formation condition of the strong interaction caused by the addition of -NH_2 in the synthesis of waterborne polyurethane dispersion in our submission. EDA was added to the reaction system in two ways. One was that EDA, which has been dissolved in 5mL of water, was added and stirred five minutes before the water emulsification. The other was that the EDA was added after the water emulsification.

The results showed that the former way produced gel, but the latter way did not produce gel. The gel produced by the former way was presumed to be due to the strong interaction between -NH_2 of the chain extender and -COOH group in MEK instead of in water.

And more, some work disclosed the strong interaction between the –COOH and -NH- or -NH2 in ethanol at room temperature.[2] Furthermore, it was reported recently that there existed the direct coupling formation of amide bond (-NH-CO-) from –COOH and –NH_2 on the graphene oxide surface [3] or on the Au(111) surface[4].

3) Another Comparative experiment was carried out to characterize the strong interaction between –NH_2 and –COOH or -COOH·$N(CH_2CH_3)_3$ We conducted FTIR analysis on the original DMPA, TEA, EDA, the product DMPA-EDA and the product DMPA-TEA-EDA. The product DMPA-EDA was obtained by mixing DMPA (0.04mol) and EDA (0.02mol) in methyl ethyl ketone (MEK) at room temperature. The product DMPA-TEA-EDA was obtained by that DMPA (0.04mol) and TEA (0.04mol) were firstly mixed well in MEK at room temperature and then mixed fully with EDA (0.02mol). The result is shown in Figure S9(a) and Figure S9(b).

In Figure S9(c), we could find the emergence of peaks at ~1666cm^{-1}, ~1621cm^{-1} and ~1560cm^{-1} in DMPA-EDA, which were absent in pure DMPA and EDA. Peaks at ~1666cm^{-1}, ~1621cm^{-1} and ~1560cm^{-1} at DMPA-EDA are quietly distant from the peak ~1682cm^{-1}(C=O) of DMPA and the peak ~1593cm^{-1} (N-H) of EDA, which means those original peaks are red-shifted and those original bonds are weakened in the product. In addition, it is known that an amide I peak located at ~1648cm^{-1} and amide II peak at ~1581cm^{-1}. The above presented that strong interaction existed between the –COOH of DMPA and the –NH_2 of EDA.

In Figure S9(d), we could find the emergence of peaks at ~1651cm^{-1}, ~1626cm^{-1} and ~1560cm^{-1} in DMPA-TEA-EDA, which were absent in pure DMPA, TEA and EDA. Peaks at ~1651cm^{-1}, ~1626cm^{-1} and ~1560cm^{-1} at DMPA-TEA-EDA are quietly distant from the peak ~1682cm^{-1}(C=O) of DMPA and the peak ~1593cm^{-1} (N-H) of EDA, which means those original peaks are red-shifted and those original bonds are weakened in the product. In addition, it is known that an amide I peak located at ~1648cm^{-1} and amide II peak at ~1581cm^{-1}. The above presented that strong interaction was still existed between the -COOH -COOH·$N(CH_2CH_3)_3$ of DMPA·TEA and the –NH_2 of EDA, although the start substance is not –COOH of DMPA.

Therefore, we concluded that the combinations of –COOH of DMPA or -COOH·$N(CH_2CH_3)_3$ of DMPA·TEA and –NH/-NH_2 lead to the gelation in PSA2 and PSA3.

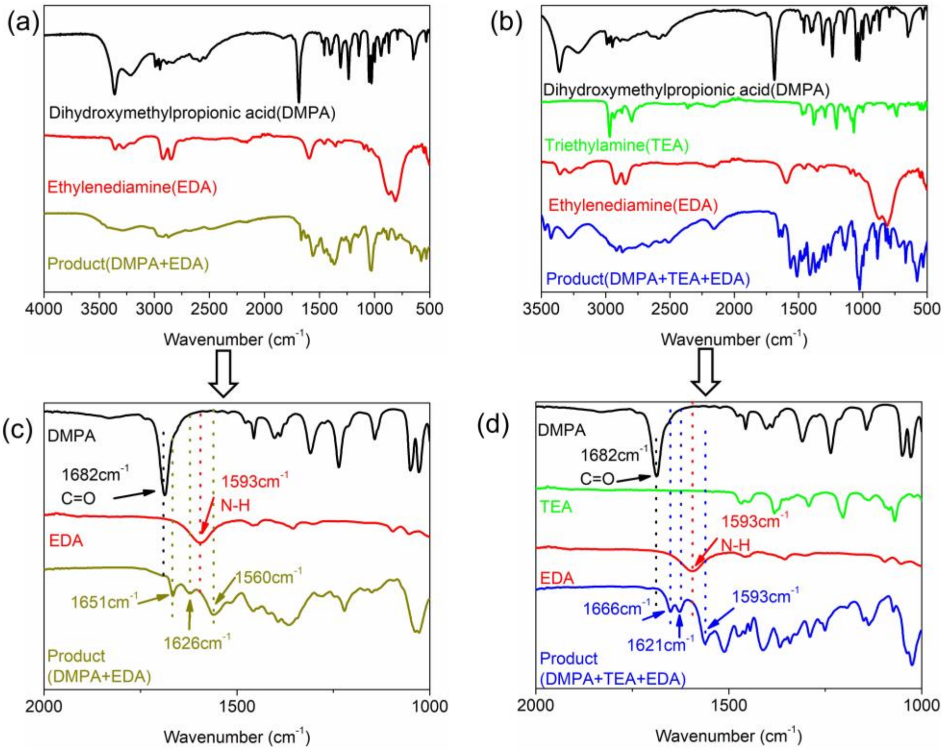

Figure S9. (**a**) Infrared spectroscopy of the reactants (EDA, DMPA) and product (DMPA-EDA) in methyl ethyl ketone. (**b**) Infrared spectroscopy of the reactants (DMPA, TEA, EDA) and product (DMPA-TEA-EDA) in methyl ethyl ketone. (**c**) and (**d**) are partial Infrared spectroscopy analysis of (a) and (b), respectively.

Table S1. Solid contents, glass transition temperature (Tg), temperature onset (T_{onset}) of thermal decomposition, and temperature of 5% ($T_{5\%}$) and 50% ($T_{50\%}$) mass loss, respectively.

Sample Code	Solid Content (wt%)	Tg (°C)	T_{onset} (°C)	$T_{5\%}$ (°C)	$T_{50\%}$ (°C)
PSA1	23.2±0.5	−51.58	206.91	276.91	376.91
PSA2	22.7±0.5	−51.97	205.13	280.12	380.12
PSA3	22.3±0.5	−52.24	205.08	272.58	380.08
PSA4	24.1±0.5	−51.94	203.15	273.12	375.65

References

1. Ge-Wen, X., *Waterborne polyurethane material*; Chemical Industry Press: Beijing, China, 2007; Vol. 12, pp. 2-3.
2. Takai-Yamashita, C.; Imabeppu, H.; Fuji, M. Synthesis of hollow silica nanoparticles using poly (acrylic acid)-3,3′-diaminodipropylamine template. *Colloids Surf. A Physicochem. Eng. Asp.* **2015**, *483*, 81–86.
3. Maslekar, N.; Zetterlund, P.B.; Kumar, P.V.; Agarwal, V. Mechanistic Aspects of the Functionalization of Graphene Oxide with Ethylene Diamine: Implications for Energy Storage Applications. *ACS Appl. Nano Mater.* **2021**, *4*, 3232–3240.
4. Yang, B.; Niu, K.; Haag, F.; Cao, N.; Zhang, J.; Zhang, H.; Li, Q.; Allegretti, F.; Bjork, J.; Barth, J.V.; et al. Abiotic Formation of an Amide Bond via Surface-Supported Direct Carboxyl-Amine Coupling. *Angew. Chem. Int. Ed. Engl.* **2022**, *61*, e202113590.
